# Case report of penile squamous cell carcinoma continuous treatment with *BRCA2* mutation

**DOI:** 10.1186/s12957-024-03305-9

**Published:** 2024-02-09

**Authors:** Qing Zhang, Yaping Li, Yanrui Zhang, Zhiping Deng, Yi Ding

**Affiliations:** 1 Department of Orthopaedic Oncology Surgery, Beijing Jishuitan Hospital, Peking University, Beijing, China; 2 Acornmed Biotechnology Co. Ltd, Beijing, China; 3 Department of Pathology, Beijing Jishuitan Hospital, Peking University, Beijing, China

**Keywords:** Penile cancer, *BRCA2* mutation, Treatment, Olaparib, Case report

## Abstract

**Background:**

Penile squamous cell carcinoma (PSCC) is a highly aggressive malignancy with a poor prognosis. *BRCA1/2* mutations are associated with impaired DNA double-strand break repair and are among the common mutations in penile cancer, potentially paving the way for poly ADP-ribose polymerase inhibitor therapy.

**Case presentation:**

We report a 65-year-old male with PSCC who progressed to thigh metastasis at 10 months after partial penectomy. Next-generation sequencing showed that the penis primary lesion and metastatic thigh lesion harboured a *BRCA2* mutation. Chemotherapy plus immunotherapy was used for treatment, and the thigh metastasis was found to involve no tumour. Progression-free survival (PFS) lasted for 8 months until the appearance of lung metastasis. Afterwards, the patient benefited from second-line therapy of olaparib with pembrolizumab and anlotinib, and his disease was stable for 9 months. The same *BRCA2* was identified in the lung biopsy. Given the tumour mutation burden (TMB, 13.97 mutation/Mb), the patient received third-line therapy with nivolumab plus ipilimumab, but PFS only lasted for 3 months, with the appearance of right frontal brain metastasis. Then, the patient was treated with radiation sequential fluzoparib therapy as fourth-line treatment, and the treatment efficacy was evaluated as PR. Currently, this patient is still alive.

**Conclusions:**

This is the first report of penile cancer with *BRCA2* mutation, receiving a combination treatment with olaparib and experiencing a benefit for 9 months. This case underscores the pivotal role of *BRCA2* in influencing treatment response in PSCC, providing valuable insights into the application of targeted therapies in managing recurrent PSCC with *BRCA2* alterations. This elucidation establishes a crucial foundation for further research and clinical considerations in similar cases.

## Background

Penile cancer is a rare disease, constituting less than 1% of male malignancies. Almost 95% of penile cancers are penile squamous cell carcinoma (PSCC) [1]. Unfortunately, patients with metastatic PSCC have poor prognosis, with a 5-year overall survival (OS) rate of only 5–10% [2]. The first option for metastatic PSCC remains platinum-based chemotherapy, but the response rate of 15–55% is disappointing [3]. After unsuccessful chemotherapy, recurrent cases are confronted with limited treatment options, mainly involving radiotherapy and immunotherapy. While some patients expressing EGFR may derive benefits from EGFR-targeted therapy, it is noteworthy that certain studies indicate a weak correlation between EGFR expression and treatment response [2]. Therefore, it is meaningful to explore multimodal management and potential targeted therapies.

Previous research on metastatic PSCC genomic profiling showed that *TP53*, *CDKN2A*, *PIK3CA*, *EGFR*, and *BRCA2* were the frequently mutated genes. Genetic testing facilitates personalized tumour treatment. For example, PI3KA-specific inhibitor and CDK4/6 inhibitor to treat patients with *PIK3CA* and *CDKN2A* mutations in advanced breast cancer [4], deleterious mutations in DNA damage responsive (DDR) genes are frequently associated with response to PARP inhibitors and platinum chemotherapy, and *BRCA1/2* are the most well-described genes in the pathway. Ali et al. reported that *BRCA2* insertions/deletions were found in 10% of 20 patients with advanced PSCC [5]. Some solid tumours with *BRCA2* mutation have been shown to be sensitive to chemotherapy, PARP inhibitors (PARPi), and immunotherapy [6]. However, effectiveness in penile cancer remains unclear, with no evidence reported to date.

Here, we report a rare case of a patient with metastatic PSCC and a somatic *BRCA2* mutation who received multiline therapy, including disease stabilization after treatment with olaparib combined with pembrolizumab and anlotinib. This patient also responded well to chemotherapy plus immunotherapy and radiotherapy, providing new choices for metastatic PSCC treatment strategies in the future.

## Case presentation

**Fig. 1:**
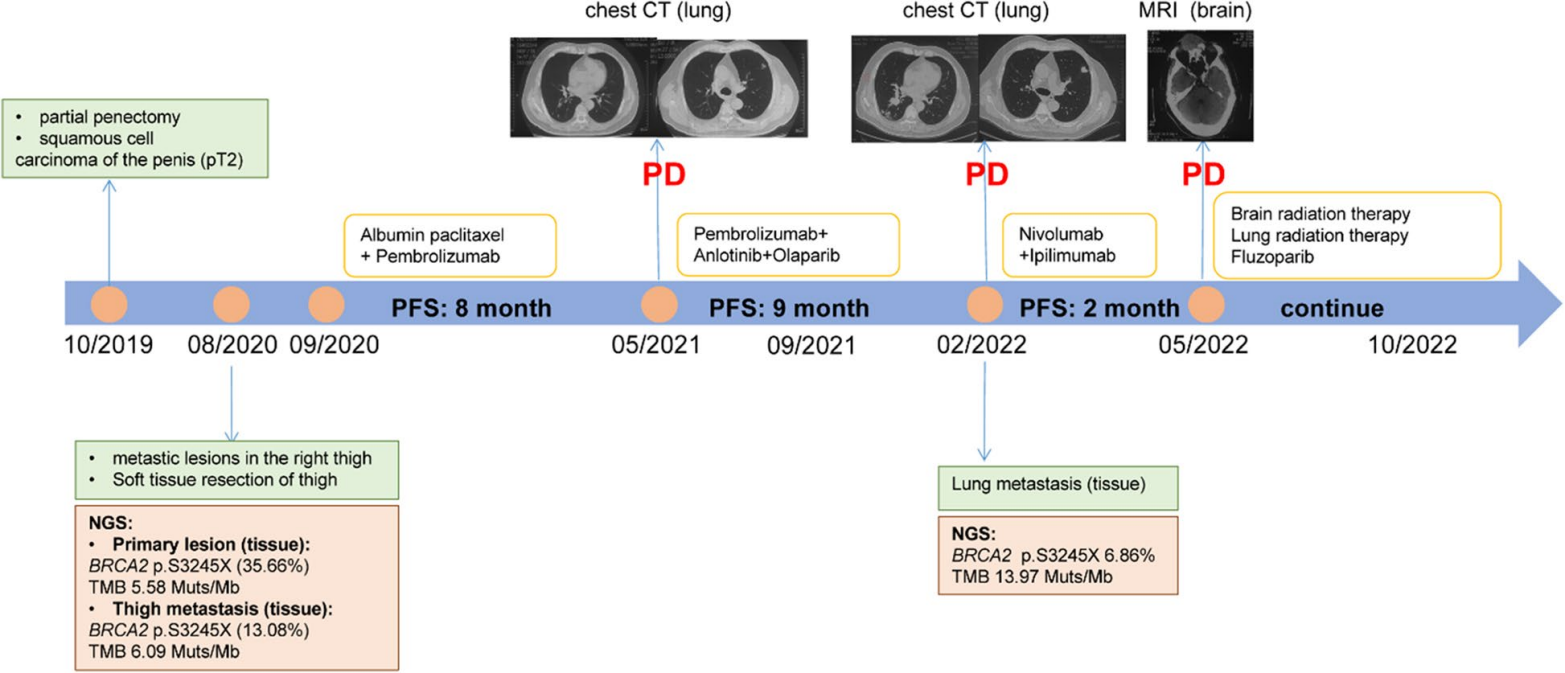
Tumour progression of the patient with mPSCC

A 65-year-old man underwent partial penectomy, and postoperative pathology revealed highly to moderately differentiated stage pT2 PSCC (AJCC eighth edition TNM staging) in October 2019. The treatment history of this patient is shown in Fig. 1.

The patient did not receive any medication after the operation. In August 2020, a follow-up examination revealed a mass in the right lower limb, and the patient was admitted to Beijing Jishuitan Hospital. Then, he underwent excision of soft-tissue tumours in the thigh under intralesional anaesthesia. Postoperative pathology showed metastatic squamous cell carcinoma, with no tumour remnants on the glass surface or skin margins. And no lymph node metastasis was observed.

With the patient’s consent, next-generation sequencing of the PSCC tissue sample (including the penis primary lesion and the metastatic thigh lesion) was performed using the Acornmed 808-gene panel (Table 1). Somatic gene mutations for the penis primary lesion were detected, including *LATS1* p.k751_A752 delinsSSCX (15.73%), *BRCA2* p.S3245X (13.08%), and *EGFR* copy number gain (7.579X). Somatic gene mutations for the metastatic thigh lesion detected included *BRCA2* p.S3245X (35.66%), *EGFR* copy number gain (6.632X), *WT1* copy number gain (6.607X), and *DNMT1* copy number gain (4.009X). The TMB for the penis primary lesion and the metastatic thigh lesion was 5.58 and 6.09 mutations/Mb, respectively. PD-L1 immunohistochemistry (IHC) (Dako 22C3 pharmDx) showed a tumour proportion score of 5% (penis primary lesion) and < 1% (metastatic thigh lesion).

**Table 1:** Summary of gene test results and mutations that may have clinical significance

Origin	Gene	Mutation	Variation frequency
Penis primary lesion	LATS1	p.k751_A752	15.73%
	BRCA2	p. S3245X	13.08%
	EGFR	Copy number gain	7.579X
Metastatic thigh lesion	WT1	Copy number gain	6.607X
	DNMT1	Copy number gain	4.009X
	BRCA2	p. S3245X	35.66%
	EGFR	Copy number gain	6.632X
Metastatic lung lesion	BRCA2	p.S3245X	6.86%
	EGFR	Copy number gain	7X

The patient was administered chemotherapy plus immunotherapy (pembrolizumab 200 mg d4; albumin paclitaxel 200 mg/100 mg d1/d5 plus cisplatin 140 mg d1) in September 2020. Eight months later, lung metastasis was detected. Chest computed tomography (CT) revealed (on May 2021) multiple nodules in the dorsal segment of the lower lobe of the right lung and irregular nodules in the anterior segment of the upper lobe of the left lung, both of which were considered possible metastasis.

**Fig. 2:**
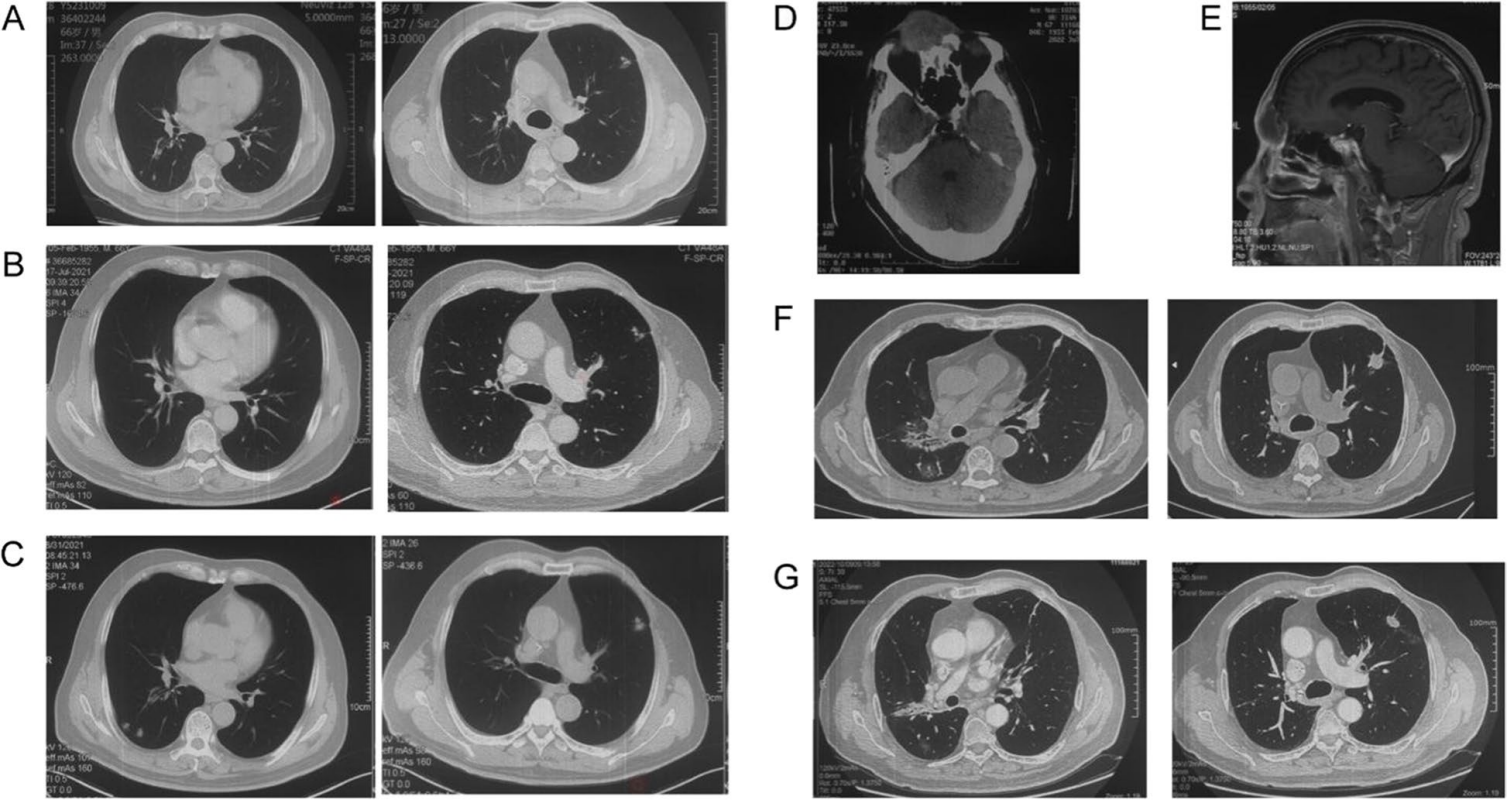
**A** Baseline CT image of lung metastases before olaparib treatment. **B** Chest CT image after 2 months olaparib treatment. **C** Chest CT image after 4 months olaparib treatment, the patient was evaluated as having stable disease. **D** Baseline MRI image of brain metastases before radiotherapy treatment. **E** MRI of brain metastases after 5 months radiation sequential fluzoparib therapy. **F** Chest CT after 3 months radiation therapy. **G** chest CT after 5 months radiation sequential fluzoparib therapy

Considering the *BRCA2* mutation, the patient was then started on olaparib plus pembrolizumab and anlotinib treatment (olaparib 300 mg (twice daily [BID]); pembrolizumab 200 mg every 3 weeks (Q3 W) and anlotinib 12 mg once a day from day 1 to 21 of a 21-day cycle) in May 2021. Several days later, his physical strength and mental status improved significantly. Follow-up CT scans after two cycles revealed that the metastasis centre was significantly reduced, and 4 months after receiving olaparib, the evaluation showed a sustained response. Due to COVID-19, the patient was not followed up until February 2022. Chest CT showed lesions in the dorsal segment of the right lower lobe and nodules in the left anterior lobe that were both increased (Fig. 2). Lung biopsy confirmed metastatic PSCC, and target sequencing detected the same *BRCA2* (6.86%) as well as *EGFR* copy number gain (7X). The TMB was calculated as 13.97 mutation/Mb.

Due to the high TMB, the patient joined a clinical trial and received dual immunotherapy (nivolumab 200 mg + ipilimumab 50 mg) in March 2022. However, he found an enlarged mass on his right forehead in May 2022. Cerebral magnetic resonance imaging revealed a space-occupying cystic lesion in the right frontal lobe with a maximal diameter of 47 mm. Subsequently, the treatment plan was changed, and he underwent stereotactic brain radiosurgery (dose per fraction 300 Gy, total dose 3000 Gy). In parallel, the patient underwent stereotactic lung radiosurgery (dose per fraction 200 Gy, total dose 5000 Gy). In August 2022, after radiotherapy treatment, a chest CT scan was performed. Considering the treatment, he began receiving fluzoparib (100 mg every day) in September 2022. Follow-up chest CT scans (October 2022) showed central lesions in the dorsal segment of the lower lobe of the right lung and central nodules in the left anterior lobe that were both significantly decreased. Brain MRI revealed significant tumour reduction, and treatment effectiveness was evaluated as PR (Fig. 2). The patient continued to receive radiotherapy.

## Discussion and conclusions

The prognosis of this patient before starting treatment was particularly poor, not only because his tumour harboured *BRCA2* mutation and *EGFR* copy number gain but also because he had multiple metastases within 1 year of diagnosis. With the typical prognosis of metastatic PSCC being less than 1 year, the 2-year survival of this patient is remarkable, especially given that he is currently alive [2]. This patient may have benefited from mutation-specific therapies such as chemotherapy plus immunotherapy, olaparib, and radiotherapy.

A previous study reported cisplatin combined with ifosfamide and paclitaxel (TIP regimen) as prior treatment for metastatic penile cancer [2]. Pagliaro et al. reported 30 patients with locally advanced disease who received neoadjuvant TIP; 15 (50.0%) reached objective response, comprising 3 complete responses (CRs) and 12 partial responses (PRs). Nineteen patients (63.3%) in this group had disease progression or recurrence [7]. Currently, there is no standard second-line regimen after first-line chemotherapy failure. In a study of 17 patients who underwent ≥ 1 salvage therapy after tumour progression from the first treatment, those who were treated with a second cisplatin-based therapy had a median overall survival (OS) of 5.6 months, and those who did not receive a second cisplatin-based therapy had a median OS of 4.3 months [8].

Given the rarity of mPSCC and the high recurrence rate after conventional treatment, it is critical to explore the genomic profile of mPSCC for potential therapeutic targets. Of 20 mPSCC patients who were enrolled to analyse comprehensive genomic profiling (CGP), 10% carried *BRCA2* mutations [5]. BRCA2 consists of 5 domains, which bind to DNA and interact with RAD51. Therefore, BRCA2 plays an important role in error-free repair of DNA double-strand breaks by mediating orderly assembly of RAD51 on ssDNA. Individuals with *BRCA2* mutations are susceptible to breast, ovarian, and other cancer types [9]. While the genomic landscape of mPSCC has been previously reported, the therapies received by patients and their responses to treatment were not given.

*BRCA1/2* mutation carriers are more responsive to chemotherapy treatments than noncarriers in breast cancer and urinary and/or ovarian cancer [10]. Recently, some studies have also shown that the immunostimulatory effects of chemotherapy can improve the efficacy of many immunotherapies because of enhanced genomic instability and immunotherapy activity [11]. and cytoplasmic dsDNA-induced DNA damage and immunogenicity have been proven to induce cell death [12]. Therefore, clinical trials have been developed to investigate the efficacy of combining chemotherapy and ICIs; for example, a significantly longer survival has been observed in non-small cell lung cancer (NSCLC) patients treated with pembrolizumab co-administered with chemotherapy as the first line [13]. Thus far, few studies have applied immune checkpoint inhibitors (ICIs) plus chemotherapy for PSCC. Li et al. reported the efficacy of immunotherapy plus chemotherapy in a patient with PSCC after recurrence. The disease progressed with multiple enlarged inguinal lymph nodes at 11 months after surgery, and immunotherapy combined with chemotherapy was administered. Pelvic magnetic resonance imaging (MRI) showed that the multiple lymph nodes in the groyne area disappeared [14]. However, no relevant reports on immunotherapy combined with chemotherapy in advanced penile SCC with *BRCA* mutations are available.

Here, we report the first mPSCC patient with *BRCA2* mutation whose disease was stable for 9 months when receiving treatment with olaparib combined with pembrolizumab and anlotinib. *BRCA2* p.S3245X was detected in the penis primary lesion, metastatic thigh lesion, and lung lesion of this patient. As with previous studies in other solid tumours, tumours with *BRCA2* mutations are sensitive to treatment with poly (ADP-ribose) polymerase inhibitors (PARPi). Olaparib is a PARPi that inhibits PARP enzymes such as PARP1, PARP2, and PARP3. PARP enzymes are critical for DNA transcription and repair [15]. Based on the results of a series of clinical trials, olaparib has been approved by the FDA as a treatment for ovarian cancer, breast cancer, pancreatic cancer, and prostate cancer [16]. Meanwhile, the FDA has specified that the mutation status of patients should be identified before treatment. To date, several biomarkers have been confirmed to be able to indicate the sensitivity of patients to olaparib, including *BRCA* mutation and homologous recombination deficiency (HRD)-positive and homologous recombination repair (HRR) gene mutation. However, olaparib usage is linked to varied adverse effects, as revealed in a meta-analysis of 14 studies with 5119 cases [17]. Adverse reactions vary across different cancers. Notably, fatigue is prominent in pancreatic cancer, while ovarian cancer shows increased severity in anaemia, neutropenia, nausea, and vomiting. Breast cancer exhibits notable grade 3 or above adverse reactions with fatigue and vomiting. Furthermore, there is currently no relevant research on the side effects of olaparib in penile cancer. Therefore, using olaparib in penile cancer carries maybe certain risks. Our case is the first report of a penile cancer patient with *BRCA* mutation benefiting from olaparib treatment, but further research is needed to expand its applicability in the future.

*BRCA1/2* mutation carriers are not only more responsive to chemotherapy and PARPi than noncarriers but are also sensitive to radiotherapy [18]. Alain Fourquet et al. reported that breast cancer patients who received radiotherapy had a major clinical response rate of 68% (13/19 tumours). This study suggests that *BRCA1/2* mutations are associated with higher response rates to radiosensitivity in breast cancers. In a recent systematic review of the literature, PARPi were also efficient radiosensitizers capable of enhancing the death ratio between 1.04 and 2.87 in several tumour models [19, 20]. The reason is the synergistic effects on DNA damage caused by ionizing radiation and inhibition of proteins essential for DNA damage repair by PARPi. Radiotherapy is used for penile cancer, but few articles have reported efficient radiotherapy. In our case, the patient carried a *BRCA2* mutation and received PARPi therapy after radiotherapy, and follow-up chest CT scans (October 2022) showed significant tumour shrinkage. These findings indicate that in penile cancer, cases involving *BRCA* mutation are sensitive to radiotherapy and/or PARPi. However, larger clinical samples may be needed to confirm this in the future.

Of note, in our case, the patient’s disease was stable at 9 months after triple therapy of olaparib with pembrolizumab and anlotinib, and the patient also benefits from radiation therapy and chemotherapy plus immunotherapy. All of these results indicate that the combination of immunotherapy and targeted therapy as well as radiation therapy were beneficial for restricting tumour progression in this patient. Therefore, to potentially increase treatment efficacy and prevent the development of disease resistance, we hypothesize that if radiation therapy is used immediately after lung metastasis, it may further improve the quality of life of patients.

In conclusion, the drugs available for treating recurrent PSCC are very limited. This is the first report of olaparib efficacy in treating PSCC with *BRCA2* mutation. We report a PSCC patient with *BRCA2* mutation who received olaparib combined with pembrolizumab and anlotinib, with satisfactory effects. Furthermore, posterior-line treatment options for recurrent PSCC with *BRCA2* mutation were studied, and the effectiveness of chemotherapy combined with immunotherapy and radiotherapy was verified. The results suggest potential treatment options for advanced or refractory PSCC and show that genetic testing facilitates personalized tumour treatment, offering a pathway to individualized therapeutic strategies.

## Data Availability

All data generated or analysed during this study are included in this article.

## Abbreviations

PSCC: Penile squamous cell carcinoma
PFS: Progression-free survival
TMB: Tumour mutation burden
OS: Overall survival
PARPi: PARP inhibitors
IHC: Immunohistochemistry
CT: Computed tomography
NSCLC: Non-small cell lung cancer
ICIs: Immune checkpoint inhibitors
MRI: Magnetic resonance imaging
HRD: Homologous recombination deficiency
HRR: Homologous recombination repair
